# An ultrasound observation study on the levator hiatus with or without diastasis recti abdominis in postpartum women

**DOI:** 10.1007/s00192-021-04783-1

**Published:** 2021-04-17

**Authors:** Peng Tian, Dong Mei Liu, Chao Wang, Yu Gu, Guo Qing Du, Jia Wei Tian

**Affiliations:** 1 Department of Ultrasonography, The Second Affiliated Hospital of Harbin Medical University, No. 246, Xuefu Road, Nanggang District Harbin, 150081 People’s Republic of China; 2 Department of Ultrasonography, The Daqing Oilfield General Hospital, Daqing, People’s Republic of China

**Keywords:** Diastasis recti abdominis, Levator hiatus, Mode of delivery, Ultrasound

## Abstract

**Introduction and hypothesis:**

We hypothesized that differences in post-partum levator hiatus (LH) measurements, as well as the area of urethra and bladder (AUB), viewed under ultrasound, correlate with diastasis rectus abdominis (DRA) occurrence. The primary objective of this study is to determine ultrasound parameters available for diagnosing DRA in post-partum women. We compared LH and AUB measurements under ultrasound in primiparous women, with and without DRA, at 24–26 weeks postpartum.

**Methods:**

One hundred ninety-four women underwent routine examination, including a self-made clinical symptoms questionnaire, DRA evaluation, and LH and AUB measurements. Independent samples *t*- and chi-squared tests were used to compare the differences between women with and without DRA.

**Results:**

DRA incidence was significantly higher among those who underwent cesarean section (CS) than for vaginal delivery (VD) (*P* = 0.038). DRA patients could potentially have urinary urgency, frequency, pain, dysuria, and perineal tears. Additionally, statistically significant differences were found between VD patients, with or without DRA, in the resting LH transverse diameter (TrD) (*P* = 0.032) and the area of the levator hiatus (ALH) (*P* = 0.048) as well as AUB at Valsalva (*P* = 0.049). No differences, however, were found between the DRA and no DRA groups for all those measurements among women who had cesarean deliveries.

**Conclusions:**

DRA was more likely in post-CS women. Furthermore, the results showed a plausible association between DRA occurrence and LH expansion, especially in women with VD under rest and Valsalva. This could be useful for developing therapeutic plans based on these parameters for post-partum rehabilitation of women with DRA to avoid long-term complications.

**Supplementary Information:**

The online version contains supplementary material available at 10.1007/s00192-021-04783-1.

## Introduction

The rectus abdominus is involved in stabilizing an individual’s trunk and pelvis, helping to maintain posture and enable them to participate in regular physical activity. Diastasis rectus abdominis (DRA) is the increase in the inner diameter of the rectus abdominis on both sides of the linea alba, which commonly results in twisted/abnormal postures [1]. DRA can possibly lead to limitations in conducting physical exercise, as well as lumbosacral and hip pain, ultimately negatively impacting the individual’s quality of life [2]. Pregnancy and delivery have been regarded as major risk factors for DRA and pelvic floor dysfunction (PFD), which is supported by a study demonstrating a relationship between DRA and support-related pelvic floor dysfunction diagnoses of stress urinary incontinence (UI), fecal incontinence, and pelvic organ prolapse (POP). However, such impacts have been contested, with some studies, such as one from Norway, finding that women with diastasis were no more likely to have weaker pelvic floor muscle strength (PFMS), UI, or POP. This finding is confirmed by research showing similar results in women at 6–8 weeks postpartum [3]. Another study likewise indicated that no significant difference was present between DRA and non-DRA groups with respect to lumbosacral pain [4, 5]. In fact, the exact relation among pregnancy, delivery, DRA, and PFD is still a controversial topic among urologists, gynecologists, and obstetricians, and some studies have found either positive or negative correlations among those factors [1].

The inner diameter of the rectus abdominis and measurement of the levator hiatus (LH) can both be easily obtained using ultrasound, a simple, highly reliable, and non-invasive method [6–8]. Vaginal delivery is strongly associated with a larger, more distensible LH and a greater degree of bladder neck mobility, both antenatally and postpartum. Cesarean section reduces postnatal symptoms but does not prevent them [9]. Due to the contradictions in current studies regarding the relation of DRA, PFD, and related symptoms, we explored whether DRA was associated with LH to clarify the relationship between intra-abdominal and pelvic pressures.

## Materials and methods

### Study subjects and determining sample size

Ethical approval was not required in this study, because the data originated from the ultrasound report management system. All post-delivery patient examinations were routine ones carried out in our hospital [10]. After delivery, women were reviewed at 42 days, 3 months, and 6 months. Gynecological examination, as well as routine blood, urine, liver, and kidney function tests, plus ultrasonography, were performed as routine examinations. This was a cross-sectional study, and data were collected from the postpartum review clinic at the Daqing Oilfield General Hospital in China from January 2018 to February 2020.

**Fig. 1:**
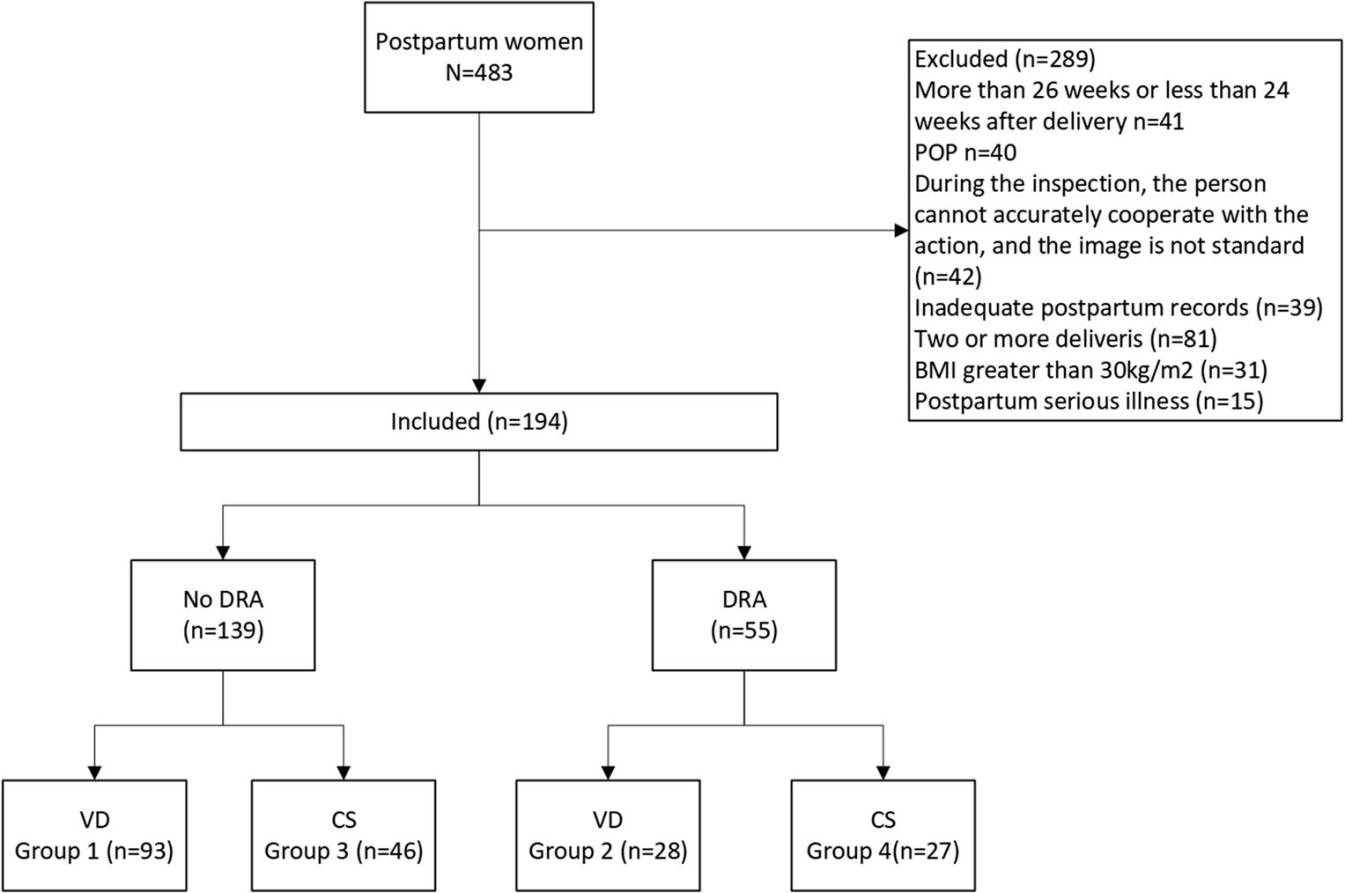
Flowchart

For this analysis, the inclusion criteria were as follows (Fig. 1): Women who underwent regular examination at the maternity clinic during pregnancy and gave birth in Daqing Oilfield General Hospital, women coming back for examinations during 24–26 weeks postpartum, women with only one childbirth, women with complete obstetric data, women with body mass index (BMI) ≤ 30 kg/m^2^, and women between the ages of 20–45 years. Four hundred eighty-three women were admitted to our hospital for postpartum follow-up during this period. Two hundred eighty-nine women were excluded because they did not come to the hospital for a 24–26 week postpartum follow-up (*n* = 41), had POP (*n* = 40), had moved incorrectly during ultrasound, yielding unclear images (*n* = 42, meaning the women did not perform the Valsalva and contraction maneuver [11]), had incomplete obstetric/postpartum records (*n* = 39), had delivered > 1 baby (*n* = 81), had a BMI > 30 kg/m^2^ (*n* = 31), or had a serious illness (*n* = 15). Statistical analyses were then performed on the remaining 194 women. The validity of using data from 194 cases in this study is confirmed via PASS software (version 15.0; (α = 0.05, β = 0.20, bilateral testing), utilizing previously published formulas, estimating that a suitable sample size for this study would be 104 [12–14].

A self-made questionnaire was designed to record postpartum females’ age at birth, body weight (kg) and height (cm) at 24–26 weeks postpartum, delivery mode, presence of lateral episiotomy or perineal tear, and the degree of urinary frequency, urgency, leakage, or dysuria. Two sonographers with respectively 12 and 20 years of ultrasound imaging experience performed all ultrasound imaging. The investigators were blinded to the participants’ identification and delivery mode. The questionnaire study was completed by the physician assistant.

### Measurement standards and data recording

A GE Voluson E8 ultrasound diagnostic device (GE Healthcare, Waukesha, WI), with a line array probe (4–12 MHz frequency) was used to measure the rectus abdominis inner diameter, except when such a diameter was too wide. In those cases, a two-dimensional probe (3.5–7.0 MHz) was utilized instead. A four-dimensional ultrasound probe (RAB4–8-D with 5–10 MHz frequency) was chosen to measure the LH as well as the angle of urethra and bladder (AUB).

### Inter-rectus distance (IRD) measurement

**Fig. 2:**
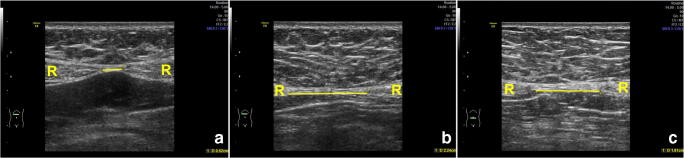
Ultrasound images of the inter-rectus distance at 5 cm above (a), 2 cm above (b), and 2 cm below (c) the umbilicus. R: Rectus abdominis. Yellow line: Measurement between the two rectus abdominis muscles at the low edge of the connection

Participants were placed in a supine resting position (knees bent 90°, feet resting on the plinth, arms alongside the body) [6, 15–17]. They were asked to perform abdominal contractions until the shoulder blades left the examination bed. The ultrasound transducer was placed transversely at three locations along the midline of the abdomen, using the center of the umbilicus as a reference: 2 cm below the umbilicus and 2 and 5 cm above the umbilicus (Fig. 2). To standardize the transducer position, each measurement location was marked with ink on the skin. All measurements were repeated three times, and the mean of the three resulting values was used. DRA diagnostic criteria are indicated below, where it was defined as a width ≥ 21 mm at 2 cm below the umbilicus, ≥ 28 mm at 2 cm above the umbilicus, or ≥ 24 mm at 5 cm above the umbilicus. Any values below those given above indicated no DRA was present in those patients [15].

We first divided the subjects into no-DRA (*n* = 139) and DRA groups (*n* = 55), according to the presence of DRA, as defined by the above parameters. The population was then further divided into four groups to further explore whether the delivery mode contributed to DRA’s influence on the size of the LH or AUB: group I (vaginal delivery [VD] with no DRA, *n* = 93), II (VD with DRA, *n* = 28), III (cesarean section [CS] with no DRA, *n* = 46), and IV (CS with DRA, *n* = 27), as shown in Fig. 1. The size of each individual group was in accordance with the estimate determined using PASS software, in which each group would have no less than 26 members. This was based upon a 50% insurance cover level of the aforementioned estimated total sample size of 104, in which a 50% sample size calculation was considered representative [18].

### Measurement of LH and AUB

Bladder emptying was confirmed by transabdominal ultrasound by evaluating the post-void residual volume, where a pathological volume was defined as > 150 ml urine. The convex volumetric ultrasound transducer was positioned trans-labially in the midsagittal plane, with patients in the lithotomy position. Measurements were performed in the axial plane of minimal hiatal dimensions [19, 20]. The ultrasound technicians performing the examinations in this study demonstrated good intra- and inter-observer reliability, as well as consistency over multiple tests, for this procedure. Moreover, the results were comparable to ones obtained through different pelvic floor assessment techniques and diagnostic tools [21]. The antero-posterior diameter (A-PD), transverse diameter (TrD), and area of levator hiatus (ALH), as well as AUB, were recorded at rest, squeeze, and Valsalva maneuver (Supplementary Fig. 1). Three-dimensional volume acquisition was required for ALH, APD, and TrD measurements [19], while 2D acquisition was necessary for AUB [20] (Supplementary Fig. 2).

### Statistical analysis

Statistical analysis was performed using IBM SPSS version 23.0. All parameters were measured three times, and the mean of those three measurements was used for analyses. Categorical variables are represented as numbers and percentages, while continuous ones are presented as mean ± standard deviations (SDs) if the data follow a normal distribution. If a normal distribution was not followed by the data, medians (interquartile ranges) were calculated and utilized instead in data analysis. Chi-square tests were performed to compare the relationship between DRA and delivery mode as well as the prevalence of various clinical symptoms associated with DRA. Independent samples *t*-test was used to analyze differences in background characteristics between women with or without DRA under the circumstances of normally distributed data. *P* < 0.05 was considered statistically significant.

## Results

The overall prevalence of DRA in our samples was 28.4%. No significant differences in maternal age, height, and BMI between women with and without DRA were found, though the DRA group had a slightly higher, but still statistically insignificant, body weight compared to those without DRA, at 64 and 60 kg, respectively. However, with respect to different delivery modes, DRA incidence in the CS group was significantly higher than in the VD group (*P* = 0.038; Table 1).

**Table 1:** Differences in clinical data for women with or without DRA

Characteristics	All subjects	No DRA	DRA	*P* value
Number (*n*)	194	139	55	
Maternal age (years)	31.2 ± 4.1	30 (28.8 to 33)	30 (28 to 34.3)	0.762
Height (cm)	163.5 ± 4.9	163 (160 to 167)	163 (162.8 to 168)	0.162
Body weight (kg)	60.5 (56.3–70)	60 (56 to 70)	64 (56.8 to 70)	0.622
BMI (kg/m^2^)	23.1 (21.0–25.4)	23.0 (21.0 to 25.4)	23.4 (20.9 to 25.3)	0.985
Delivery mode (*n*, %)				0.038*
VD		93 (73.2)	28 (26.8)	
CS		46 (63.0)	27 (37.0)	

*BMI* body mass index, *VD* vaginal delivery, *CS* cesarean section, *No DRA* without diastasis recti abdominis,

*DRA* diastasis recti abdominis, *P < 0.05

Using our simple self-made questionnaire, we found that a significant difference was present with respect to the occurrence of perineal tear between the DRA and no-DRA groups, where it was more common in the former (*P* = 0.005; Table 2).

**Table 2:** Clinical symptoms in women with or without DRA

	All subjects	No DRA	DRA	*P* value
Perineal-lateral incision (*n*, %)				
Yes	54 (27.8)	42 (30.2)	12 (21.8)	0.240
No	140 (72.2)	97 (69.8)	43 (78.2)	
Perineal tear (*n*, %)				
Yes	15 (7.7)	6 (4.3)	9 (16.4)	0.005*
No	179 (92.3)	133 (95.7)	46 (83.6)	
Drink coffee/strong tea (*n*, %)				
Yes	3 (1.6)	3 (2.2)	0 (0)	0.272
No	191 (98.4)	136 (98.0)	55 (100)	
Urinary frequency (*n*, %)				
No	144 (74.2)	107 (77.0)	37 (67.3)	0.273
Occasionally	44 (22.7)	29 (20.9)	15 (27.3)	
Usually	6 (3.1)	3 (2.2)	3 (5.5)	
Urine leaking (*n*, %)				
No	126 (65.0)	91 (65.5)	35 (63.6)	0.065
Occasionally	58 (29.9)	44 (31.7)	14 (25.5)	
Usually	10 (5.2)	4 (2.9)	6 (10.9)	
Difficult urination (*n*, %)				
Yes	4 (2.1)	4 (2.9)	0 (0)	0.204
No	190 (97.9)	135 (97.1)	55 (100)	

“Occasionally” refers to ≤ 3 times a week. “Usually” refers to > 3 times a week, *P < 0.05

Table 3 shows the relationship between the different delivery modes and clinical symptom prevalence with respect to the above-mentioned groups I–IV. The difference with respect to perineal tear was still present even when delivery mode was taken into account, where it was more prevalent among group II (VD with DRA) than group I (VD, no DRA) (*P* = 0.000). Higher percentages were found for group IV (CS with DRA) compared to Group III (CS, no DRA) with respect to urinary frequency, being 25.9% for “occasional” and 3.7% for “usual” occurrences in group IV versus 8.7% and 0% respectively in group III (*P =* 0.014 for “occasional/usual”). Notably, perineal tearing and perineal-lateral incision are not applicable to cesarean sections owing to the nature of the procedure.

**Table 3:** Clinical symptoms for the four groups of women

	Vaginal delivery		*P*	Cesarean delivery		*P*
Variable	Group I (*n* = 93)	Group II (*n* = 28)		Group III (*n* = 46)	Group IV (*n* = 27)	
Perineal-lateral incision (*n*, %)			0.831			
Yes	42 (45.2)	12 (42.9)				
No	51 (54.8)	16 (57.1)				
Perineal tear (*n*, %)			0.000^*^			
Yes	6 (6.5)	9 (32.1)				
No	87 (93.6)	19 (67.9)				
Drink coffee/strong tea (*n*, %)			–			0.180
Yes	0 (0)	0 (0)		3 (6.5)	0 (0)	
No	93 (100)	28 (100)		43 (93.5)	27 (100)	
Urinary frequency (*n*, %)			0.433			0.014^*^
No	65 (69.9)	18 (64.3)		42 (91.3)	19 (70.4)	
Occasionally	25 (26.9)	8 (28.6)		4 (8.7)	7 (25.9)	
Usually	3 (3.2)	2 (7.1)		0 (0)	1 (3.7)	
Urine leaking (*n*, %)			0.246			0.178
No	52 (55.9)	15 (53.6)		39 (84.8)	20 (74.1)	
Occasionally	37 (39.8)	8 (28.6)		7 (15.2)	6 (22.2)	
Usually	4 (4.3)	5 (17.9)		0 (0)	1 (3.7)	
Difficult urination (*n*, %)			0.340			0.447
Yes	3 (3.2)	0 (0)		1 (2.2)	0 (0)	
No	90 (96.8)	28 (100)		45 (97.8)	27 (100)	

Group I: no DRA and VD; group II: DRA and VD; group III: no DRA and CS; group IV: DRA and CS

Table 4 shows no significant difference being found for the LH measurements of TrD, A-PD, and ALH, as well as for AUB among all women included in this study with and without DRA. However, Supplementary Table 1 shows a statistically significant increase only for resting state TrD (*P* = 0.032) and ALH (*P* = 0.048) values between groups I and II. No statistically significant changes were present for any parameters measured in groups III and IV, no matter whether the state was rest, squeeze, or Valsalva.

**Table 4:** Measurements of the levator hiatus and angle of the bladder and urethra in women with or without DRA

Measurement status	Measuring section	No DRA (*n* = 139)	DRA (*n* = 55)	*P*
Rest	TrD (cm)	4.1 (3.8 to 4.3)	4.0 (3.7 to 4.5)	0.626
	A-PD (cm)	5.1 (4.6 to 5.5)	5.2 (4.6 to 5.6)	0.882
	ALH (cm^2^)	13.6 (11.9 to 15.9)	14.5 (11.9 to 15.8)	0.487
	ABU (degrees)	117.3 (106.5 to 126.5)	115.7 (104.8 to 132.1)	0.632
Squeeze	TrD (cm)	3.8 (3.5 to 4.1)	3.8 (3.5 to 4.1)	0.939
	A-PD (cm)	4.4 (4.0 to 4.8)	4.2 (3.8 to 4.7)	0.183
	ALH (cm^2^)	11.2 (9.7 to 13.0)	10.8 (9.8 to 12.7)	0.629
	ABU (degrees)	116.1 (108.9 to 126.1)	117.0 (107.3 to 125.0)	0.665
Valsalva	TrD (cm)	4.8 (4.3 to 5.4)	4.7 (4.3 to 5.3)	0.823
	A-PD (cm)	6.3 (5.5 to 7.1)	6.0 (5.3 to 7.0)	0.518
	ALH (cm^2^)	22.9 (18.2 to 28.5)	22.2 (16.9 to 28.0)	0.829
	ABU (degrees)	147.3 (130.9 to 160.4)	154.1 (133.6 to 166.1)	0.108

*TrD* transverse diameter of the levator hiatus at the level of the pubovaginalis; *A-PD* antero-posterior diameter of the levator hiatus;

*ALH* area of the levator hiatus; *ABU* angle of the bladder and urethra; *P < 0.05

When comparing between groups I and III as well as groups II and IV in Supplementary Table 2, to determine the presence of significant differences based on delivery mode, such differences were found between groups I and III for TrD (*P* = 0.005) and ALH (*P* = 0.035) at rest in the form of decreases. This was also the case for groups II and IV (respectively *P* = 0.007 and *P =* 0.021) as well as for A-PD at rest (*P =* 0.006). Significant decreases in TrD (*P =* 0.005), A-PD (*P =* 0.001), and ALH (*P* = 0.005) were also found between groups II and IV under squeeze conditions. As for Valsalva, TrD decreased significantly, while A-PD increased significantly between groups II and IV (respectively *P =* 0.002 and *P =* 0.008).

## Discussion

Our study has revealed a number of interesting findings with respect to the occurrence of DRA as well as its connections to delivery mode, symptoms, and levator hiatus measurements. First, DRA more frequently occurs in CS patients compared to VD. It is also more associated with perineal tears overall, and particularly in VD patients. Both these findings could respectively result from the direction of the contractive forces from abdominal and uterine muscles; during vaginal delivery, these forces are directly oriented toward the infant within the uterus to ensure delivery. This leads to rectus abdominis muscle extrusion and increased intraperitoneal pressure, yielding DRA and perineal tears. CS patients, by contrast, have more prevalent associations between urinary frequency and DRA, possibly due to complications from surgery associated with cesarean section. With respect to levator hiatus measurements, such as TrD, A-PD, ALH, and AUB, overall, the presence or absence of DRA has no significant impact. However, a micro-analysis revealed that VD patients with DRA have significant differences in TrD and ALH at rest, as well as AUB at Valsalva, compared to those without DRA, which may stem from overstretching of the pelvic floor muscles, as well as bladder squeezing, during delivery. Furthermore, significant differences in TrD and ALH are seen when comparing VD and CS without DRA at rest. More differences are present between VD and CS with DRA for TrD, A-PD, and ALH at rest and squeeze states as well as for only TrD and A-PD at Valsalva. All these findings could stem from the levator hiatus and other abdominal muscles being less stretched during cesarean section compared to vaginal delivery, explaining the smaller dimensions for most of the aforementioned parameters (TrD, ALH, AUB, A-PD); coupled with possible scar formation at the diastasis rectus abdominis post-surgery, this may yield greater susceptibility to DRA.

PFD severely impacts quality of life in women and is heavily influenced by pregnancy and delivery [22] . Its relationship with DRA and PFD is a major topic of discussion among urologists, gynecologists, and obstetricians. The present study therefore factored various covariates into the analysis, including delivery mode, as well as interactions among POP, UI, and pelvic pain with DRA, which have been studied by other researchers [4, 10].

Because the rectus abdominis diameter is influenced by pelvic muscle and abdominal external oblique muscle contractions, it is no surprise that the occurrence of DRA was affected by the delivery mode [23]. Our findings showed that women with a history of cesarean section were more prone to develop DRA, where the CS group had an incidence rate of 37.0% versus 26.8% in the VD group. The overall incidence, as determined from the clinical data, was 28.4%. All these discoveries were compatible with previous studies [9, 10], where the literature indicated a DRA rate of 35–39% for CS patients and a lower rate among VD patients. It should be noted, however, that DRA occurrence during the pregnancy and post-partum periods can vary, though the latter typically entails a progressive post-delivery decrease in the rectus abdominis inner diameter, albeit without ever vanishing [24]. The lower occurrence of diastasis during vaginal delivery could be caused by the contractive muscle force of the abdominal muscle groups being concentrated inward, directly on the uterus, to ensure the delivery of the infant. This was simultaneously coupled with the concentric extrusion of the rectus abdominis muscle, thus decreasing its inner diameter. Overall, the results from the present study were consistent with similar ones, such as the study conducted by Qing Wang et al. [10].

Traditionally, DRA is expected to more easily occur among leaner women because of the protective effects bestowed by abdominal wall fat. However, our study found that the body weight among the DRA group was slightly higher than for those with no-DRA (Table 1). This may be the result of our data not including individuals with BMI > 30 kg/m^2^, which is supported by the lack of any statistically significant difference in BMI among the women tested between the DRA and no DRA groups, as they were both around 23 kg/m^2^.

Our data also showed that perineal tearing is more likely to occur in the DRA and VD group. Perineal tear was one of the risk factors identified for some diseases, and the question of whether DRA causes perineal tearing, or vice versa, is worth further study [25]. One possibility for the connection between vaginal delivery and perineal tearing could be the inward contraction of abdominal muscles, particularly uterine, during delivery. Upon reaching a certain level of intraperitoneal pressure, greater than what the muscles can bear, the intraabdominal pressure expands outwards to find a “weak spot” (the levator hiatus and perineal body [26]) in order to deliver the fetus, if it has not emerged yet. Under such circumstances, the abdominal-pelvic cavity is not ready for delivery, which might cause the rectus abdominis diastasis and perineal tear. This connection is also supported by lower linea alba stiffness in the DRA group, according to the literature [27].

The occurrence of certain lower urinary tract symptoms, such as urinary frequency and usually having urine leakage, is more prevalent in DRA than in no DRA patients in terms of patient percentages. However, no statistically-significant increases were present when accounting for all cases in both the urinary frequency and leakage categories.

When the delivery mode is not considered, no statistically significant differences were present between DRA and no DRA patients. However, if delivery mode was accounted for, statistically significant differences were found between VD without DRA versus with DRA in regard to TrD and ALH at rest, as well as AUB at Valsalva. These differences were not found between CS patients with and without DRA, indicating that different delivery modes may affect levator hiatus measurements under DRA conditions. On the other hand, even without significant differences, most levator hiatus measurements for the DRA group are larger than for those with no DRA, possibly indicating an indirect effect between DRA and POP.

Our study was limited by the modest sample size, as well as observation biases, owing to the patients requiring strict and regular screening. Additionally, future studies would need to include pre-pregnancy information, as well as other childbirth-related factors, such as large birth weight babies [5], in order to exclude the confounding factors in relation to the occurrence of DRA and the delivery method. It is also worth noting that the follow-up stopped at 6 months post-partum but morphological changes related to pregnancy may continue to evolve well beyond that timeline. Therefore, some of these changes might have been temporary.

In conclusion, DRA and PFD are of clinical relevance, in that the occurrence of DRA varies with delivery mode, where women with cesarean section may be more susceptible to DRA. Furthermore, DRA patients were more likely to have urological symptoms and larger levator hiatus measurements.

## Supplementary Information

ESM 1 (DOCX 22 kb)

ESM 2 (PNG 493 kb)

High-resolution image (TIFF 1862 kb)

ESM 3 (PNG 888 kb)

High-resolution image (TIFF 2773 kb)

## Declarations

Written informed consent was obtained from the patients for publication of the images in Urogynecology.
