# Supplementary material for: An ultrasound observation study on the levator hiatus with or without diastasis recti abdominis in postpartum women
Source: Int Urogynecol J. 2021 Apr 17;32(7):1839–46. doi: 10.1007/s00192-021-04783-1 (PMC8295084; doi:10.1007/s00192-021-04783-1)
Supplement: Supplementary file 1 — (DOCX 22 kb) [file 192_2021_4783_MOESM1_ESM.docx]

**Supplementary Information**

for

**An** **ultrasound observation study on levator hiatus, with or without diastasis recti abdominis in postpartum women**

Peng Tian^1#,2^, Dong Mei Liu^1#^, Chao Wang^1^, Yu Gu^1^, Guo Qing Du^1*^, Jia Wei Tian^1*^

^1^Department of Ultrasonography, The Second Affiliated Hospital of Harbin Medical University, Harbin, PR China

^2^Department of Ultrasonography, The Daqing Oilfield General Hospital, Daqing, PR China

^#^Both authors contributed equally to this work

**Supplementary Figure1**. Ultrasound images of the levator hiatus at rest (A), squeeze (B), Valsalva (C) of the axial plane. A：A: anal;B: bladder ;R:rectum；S：symphysiss pubis； U: urethra; V:vagina.B：The two white lines point to levator ani.C：1)The red line： measurement of transverse diameter (TrD), 2)The yellow line：measurement of antero-posterior diameter(A-PD), 3)The blue line：measurement of area of levator hiatus (ALH).

**Supplementary Figure2**.Ultrasound images of the angle of bladder and urethra at rest (A),squeeze (B), and Valsalva (C) at the mid-sagittal plane.White line of the angle: Measurement of the angle of bladder and urethra from the urethra to the post wall of the bladder to the urethra . A: anal;B: bladder ;R:rectum；S：symphysiss pubis； U: urethra; V:vagina.

**Supplemental Table 1** Measurements of levator hiatus and the angle of bladder and urethra in women, with or without DRA, for VD or CS

| Measurement status | Measuring section | VD | | | CS | | |
| --- | --- | --- | --- | --- | --- | --- | --- |
|  |  | Group I (n=93) | Group II (n=28) | *P* | Group III (n=46) | Group IV (n=27) | *P* |
| Rest | TrD (cm) | 4.1 (3.9 to 4.3) | 4.4 (3.9 to 4.7) | 0.032* | 3.8 (3.6 to 4.2) | 3.8 (3.5 to 4.2) | 0.826 |
|  | A-PD (cm) | 5.3 (4.8 to 5.7) | 5.2 (4.7 to 5.6) | 0.501 | 4.8 (4.4 to 5.1) | 4.9 (4.3 to 5.1) | 0.454 |
|  | ALH (cm^2^) | 14.7 (12.8 to 16.8) | 15.1 (13.7 to 18.2) | 0.048* | 12.2 (10.2 to 14.3) | 12.4 (11.3 to 14.1) | 0.329 |
|  | ABU (°) | 116.4 (107.0 to 125.2) | 115.7 (104.2 to 132.1) | 0.725 | 117.5 (105.7 to 126.8) | 109.7 (100.1 to 125.3) | 0.507 |
| Squeeze | TrD (cm) | 3.9 (3.7 to 4.2) | 3.9 (3.7 to 4.4) | 0.078 | 3.6 (3.3 to 3.9) | 3.6 (3.3 to 4.0) | 0.776 |
|  | A-PD (cm) | 4.5 (4.2 to 4.9) | 4.4 (4.1 to 4.8) | 0.964 | 4.1 (3.8 to 4.6) | 4.2 (3.8 to 4.4) | 0.318 |
|  | ALH (cm^2^) | 12.2 (10.6 to 14.3) | 12.2 (10.7 to 15.6) | 0.209 | 10.1 (8.8 to 12.5) | 10.4 (9.1 to 11.1) | 0.745 |
|  | ABU (°) | 115.9 (110.2 to 128.5) | 120.8 (113.3 to 125.8) | 0.595 | 115.9 (107.8 to 124.7) | 112.3 (101.9 to 120.9) | 0.82 |
| Valsalva | TrD (cm) | 5.2 (4.7 to 5.7) | 4.9 (4.6 to 5.5) | 0.755 | 4.3 (3.8 to 4.8) | 4.4 (4.0 to 5.1) | 0.312 |
|  | A-PD (cm) | 6.7 (6.2 to 7.4) | 6.0 (5.3 to 7.6) | 0.486 | 5.9 (4.7 to 6.3) | 6.0 (5.0 to 6.7) | 0.327 |
|  | ALH (cm^2^) | 26.6 (21.5 to 30.1) | 22.9 (18.7 to 29.1) | 0.695 | 17.7 (14.3 to 21.9) | 20.7 (16.1 to 24.5) | 0.106 |
|  | ABU (°) | 152.8 (130.8 to 161.0) | 160.4 (139.1 to 178.1) | 0.049* | 141.0 (128.7 to 156.4) | 148.2 (125.5 to 162.0) | 0.137 |

Group I: No DRA and VD; Group II: DRA and VD; Group III: No DRA and CS; Group IV: DRA and CS, TrD: Transverse diameter of levator hiatus at the level of pubovaginalis; A-PD: Antero-posterior diameter of levator hiatus; ALH: Area of levator hiatus; ABU: Angle of bladder and urethra, **P*<0.05

**Supplemental Table 2** Measurements of levator hiatus and the angle of bladder and urethra in women who have undergone VD or CS, with or without DRA

| Measurement status | Measuring section | No DRA | | | DRA | | |
| --- | --- | --- | --- | --- | --- | --- | --- |
|  |  | Group I (n=93) | Group III (n=28) | *P* | Group II (n=46) | Group IV (n=27) | *P* |
| Rest | TrD (cm) | 4.1 (3.9 to 4.3) | 3.8 (3.7 to 4.2) | 0.005* | 4.4 (3.9 to 4.7) | 3.8 (3.5 to 4.2) | 0.007* |
|  | A-PD (cm) | 5.3 (4.8 to 5.7) | 4.8 (4.4 to 5.1) | 0.17 | 5.2 (4.7 to 5.6) | 4.9 (4.3 to 5.1) | 0.006* |
|  | ALH (cm^2^) | 14.7 (12.8 to 16.8) | 12.2 (10.2 to 14.3) | 0.035* | 15.1 (13.7 to 18.2) | 12.4 (11.3 to 14.1) | 0.021* |
|  | ABU (°) | 116.4 (107.0 to 125.2) | 117.5 (105.7 to 126.8) | 0.757 | 115.7 (104.2 to 132.1) | 109.7 (100.1 to 125.3) | 0.44 |
| Squeeze | TrD (cm) | 3.9 (3.7 to 4.2) | 3.6 (3.3 to 3.9) | 0.231 | 3.9 (3.7 to 4.4) | 3.6 (3.3 to 4.0) | 0.005* |
|  | A-PD (cm) | 4.5 (4.2 to 4.9) | 4.1 (3.8 to 4.6) | 0.292 | 4.5 (4.1 to 4.8) | 4.2 (3.8 to 4.4) | 0.001* |
|  | ALH (cm^2^) | 12.2 (10.6 to 14.3) | 10.1 (8.8 to 12.5) | 0.148 | 12.2 (10.7 to 15.6) | 10.4 (9.1 to 11.1) | 0.005* |
|  | ABU (°) | 115.9 (110.2 to 128.5) | 115.9 (107.8 to 124.7) | 0.272 | 120.8 (113.3 to 125.8) | 112.3 (101.9 to 120.9) | 0.642 |
| Valsalva | TrD (cm) | 5.2 (4.7 to 5.7) | 4.3 (3.8 to 4.8) | 0.051 | 4.9 (4.6 to 5.5) | 4.4 (4.0 to 5.1) | 0.002* |
|  | A-PD (cm) | 6.7 (6.2 to 7.4) | 5.9 (4.7 to 6.3) | 0.58 | 6.0 (5.3 to 7.6) | 6.0 (5.0 to 6.7) | 0.008* |
|  | ALH (cm^2^) | 26.6 (21.5 to 30.1) | 17.7 (14.3 to 21.9) | 0.23 | 22.9 (18.7 to 29.1) | 20.7 (16.1 to 24.5) | 0.713 |
|  | ABU (°) | 152.8 (130.8 to 161.0) | 141.0 (128.7 to 156.4) | 0.101 | 160.4 (139.1 to 178.1) | 148.2 (125.5 to 162.0) | 0.414 |

**P*<0.05
