# Supplementary figures and images for: An ultrasound observation study on the levator hiatus with or without diastasis recti abdominis in postpartum women
Source: Int Urogynecol J. 2021 Apr 17;32(7):1839–46. doi: 10.1007/s00192-021-04783-1 (PMC8295084; doi:10.1007/s00192-021-04783-1)

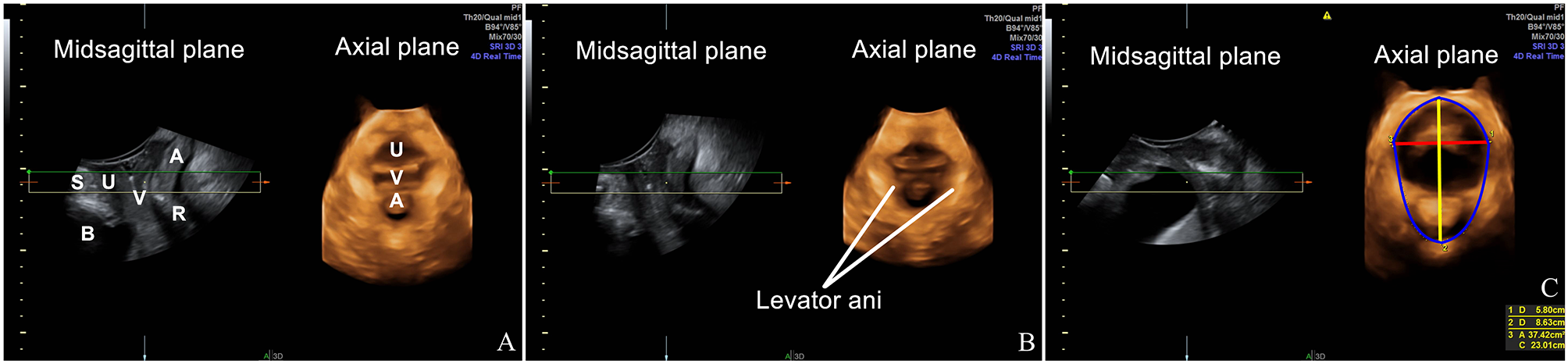

Supplement: Supplementary file 2 — (PNG 493 kb) [file 192_2021_4783_Fig3_ESM.png]

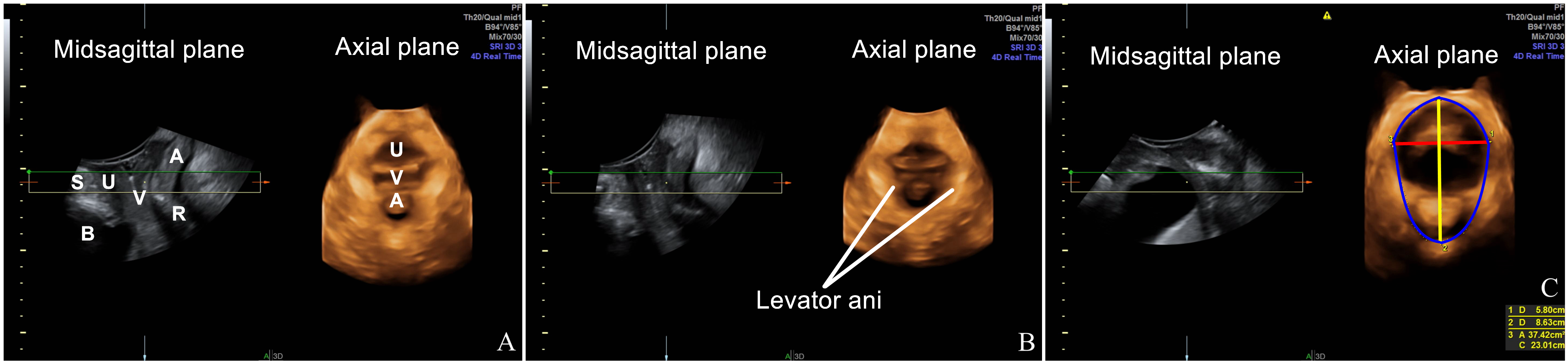

Supplement: Supplementary file 3 — High-resolution image (TIFF 1862 kb) [file 192_2021_4783_MOESM2_ESM.tiff]

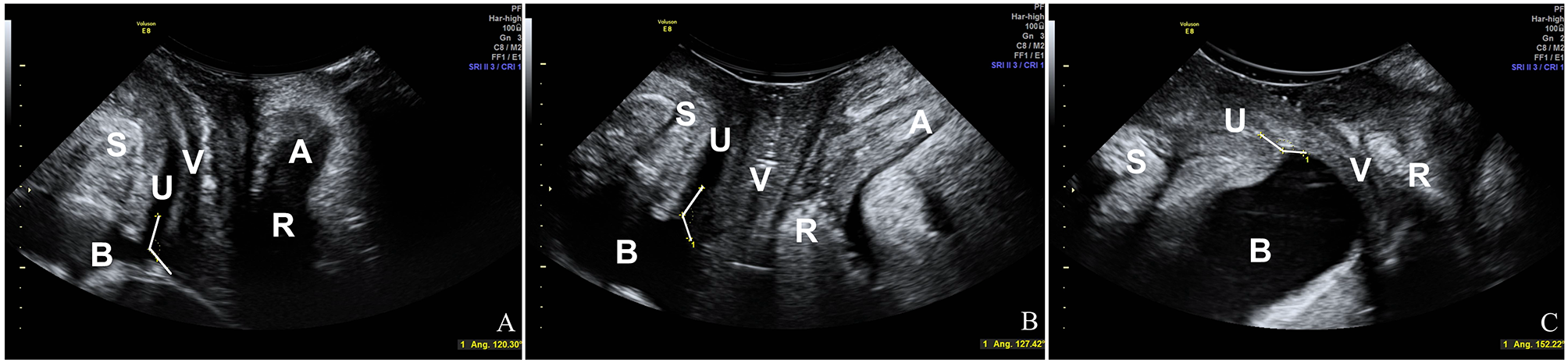

Supplement: Supplementary file 4 — (PNG 888 kb) [file 192_2021_4783_Fig4_ESM.png]

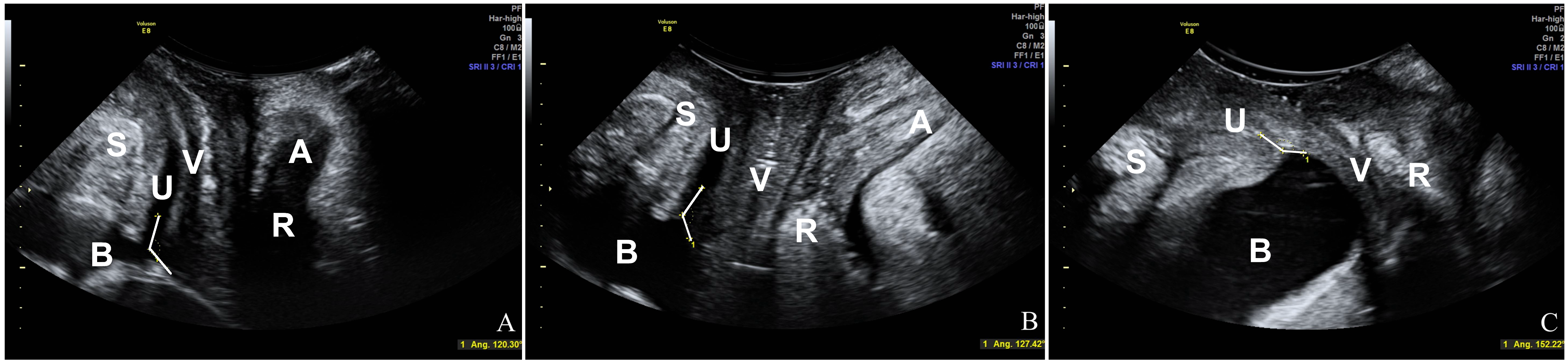

Supplement: Supplementary file 5 — High-resolution image (TIFF 2773 kb) [file 192_2021_4783_MOESM3_ESM.tiff]
